# The effect of human mobility and control measures on traffic safety during COVID-19 pandemic

**DOI:** 10.1371/journal.pone.0243263

**Published:** 2021-03-08

**Authors:** Jie Zhang, Baoheng Feng, Yina Wu, Pengpeng Xu, Ruimin Ke, Ni Dong

**Affiliations:** 1 School of Transportation and Logistics, Southwest Jiaotong University, Chengdu, China; 2 National United Engineering Laboratory of Integrated and Intelligent Transportation, Chengdu, China; 3 Department of Civil, Environmental and Construction Engineering, University of Central Florida, Orlando, FL, United States of America; 4 Department of Civil Engineering, University of Hong Kong, Hong Kong, China; 5 Department of Civil and Environmental Engineering, University of Washington, Seattle, WA, United States of America; Tongii University, CHINA

## Abstract

As mobile device location data become increasingly available, new analyses are revealing the significant changes of mobility pattern when an unplanned event happened. With different control policies from local and state government, the COVID-19 outbreak has dramatically changed mobility behavior in affected cities. This study has been investigating the impact of COVID-19 on the number of people involved in crashes accounting for the intensity of different control measures using Negative Binomial (NB) method. Based on a comprehensive dataset of people involved in crashes aggregated in New York City during January 1, 2020 to May 24, 2020, people involved in crashes with respect to travel behavior, traffic characteristics and socio-demographic characteristics are found. The results show that the average person miles traveled on the main traffic mode per person per day, percentage of work trip have positive effect on person involved in crashes. On the contrary, unemployment rate and inflation rate have negative effects on person involved in crashes. Interestingly, different level of control policies during COVID-19 outbreak are closely associated with safety awareness, driving and travel behavior, and thus has an indirect influence on the frequency of crashes. Comparing to other three control policies including emergence declare, limits on mass gatherings, and ban on all nonessential gathering, the negative relationship between stay-at-home policy implemented in New York City from March 20, 2020 and the number of people involved crashes is found in our study.

## 1. Introduction

Assessing mobility and safety impacts on the transportation system is primarily concern for policy decision-makers when an unplanned event occurs. Mobility analysis is used to identify how people change their travel behavior and safety analysis focus on how crash frequencies and severity may change from before to after an event. As vividly illustrated in the current COVID-19 pandemic, related control policy e.g. social distancing orders have had dramatic impacts on efficiency and safety of transportation system. Recently researchers and practitioners have increasingly provided a more proactive approach to evaluate the impact of COVID-19 on transportation system analysis e.g., travel patterns, speed, travel volume, vehicle mile travel and transportation performance etc. [1–5].

And nowadays the wide availability of mobile sensors has presented us with an opportunity of potentially evaluating the performance and mobility changes to the transportation systems in nearly real time. Researchers have also measured the impact of the COVID-19 on human mobility by using the mobile device location data public available from many companies such as Google, Facebook and Cuebiq which is very helpful to asset for the change of human mobility under the different control policy e.g., mass gathering limits and social distancing [4–9]. For example, the interactive COVID-19 analysis platform from university of Maryland monitors the mobility and social distancing trends by daily feeds of mobile device location data in the United States.

[7–9] The platforms produce aggregated daily mobility metrics including trip purpose, travel mode and social-demographics imputation. The integration of mobility, safety, and behavior data related to COVID-19 is expected to provide valuable insights for policy decision-makers.

**Fig 1:**
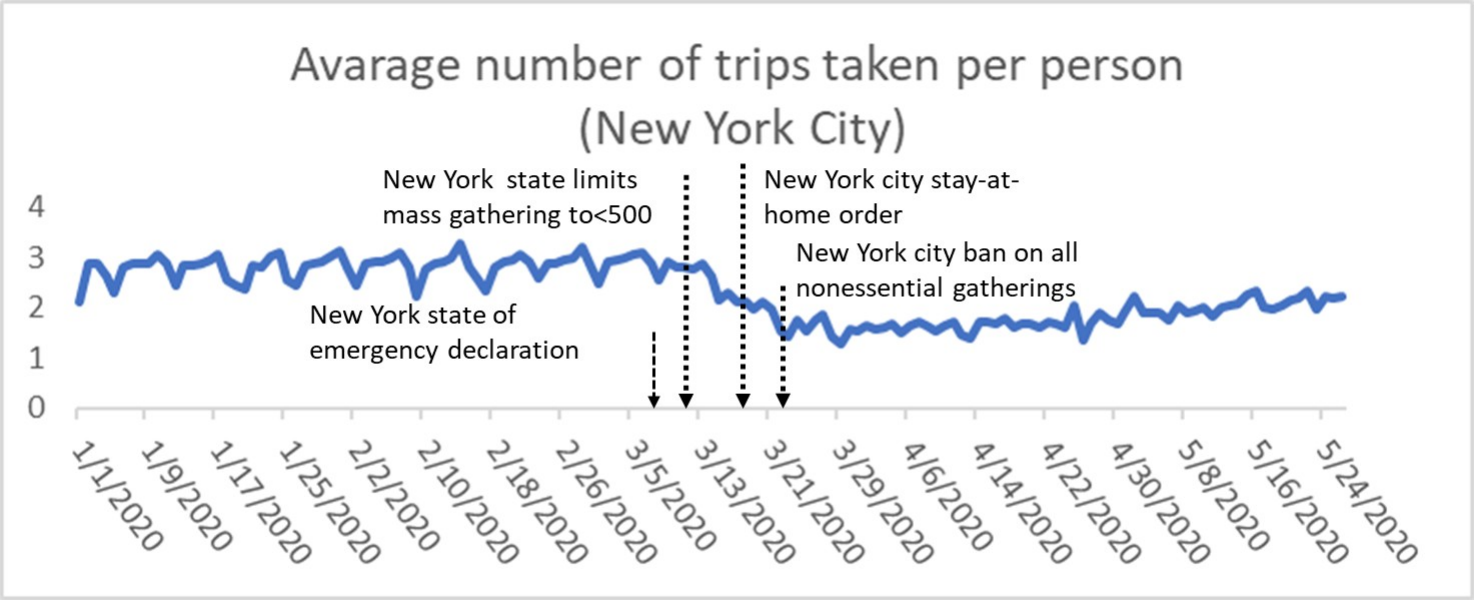
Average number of trips taken per person in New York City during January 1, 2020 and May 24, 2020.

In the case of COVID-19, the “stay-at-home” order and implications of social distancing measures, which aim to “flatten the curve” of the spread and in turn, have significantly changed people’s travel behavior in different ways. For example, there are two kinds of public policies ordering COVID-19 mitigation intervention in NYC before March. 12th, 2020 in NYC including state declaration of emergency and state limits on mass gathering. And maybe the reason for the decreasing on the average number of trips taken per person is related to these two policies implementation (see Fig 1). Some people have safety concerns of using public transit or other shared mode and avoids gathering in crowded areas [10]. With the reopen strategies implemented due to the mitigation of COVID-19, the changed behaviors may exhibit inertia [11, 12], which could be defined by the travelers continue to behave according to the social distancing order due to residual fears. However, their potential traffic safety impact should not be ignored due to the change of travel trend under the social distancing. In other words, traffic has sharply decreased and residents in a region may alter their travel patterns due to the control policies of the COVID-19, but how do residents’ travel pattern influence road safety? What behaviors are the significant contributing factors?

To investigate the impact of control policy changes on road safety during the COVID-19, we developed Negative Binomial model to evaluate the number of person involved in crashes using mobility and crash data in New York city, while adjusting for the effects of a range of confounders, such as residents’ trips, social distance policy change, percentage out of city trip and social economic factors, etc. Based on a comprehensive dataset of crash data of New York city between January 1, 2020 and May 24, 2020, this study specifically aims to assess road safety with respect to travel behavior, traffic characteristics and socio-demographic characteristics with the change of control policies for the COVID-19.

## 2. Data

### 2.1 Study area

In this study, the New York City was selected as the study area, where is the one of the most populous cities and the most densely populated major city in the United States [13]. It has 8,336,817 population distributed over about 302.6 square miles (784 km^2^) and five boroughs including Brooklyn, Queens, Manhattan, Staten Island and the Bronx.

### 2.2 Mobility data

We collected the mobility data from the interactive COVID-19 mobility impact and social distancing analysis platform [1, 2]. The COVID-19 Impact Analysis Platform currently contains travel trend metrics at the state and county levels in the United States with daily updates unless otherwise specified, which were calculated by mobile device location dataAnd other social and economics daily data source is from the National Household Travel Survey and American Community Survey.

The mobility metric data including mode imputation, trip imputation and socio demographic was used for analysis in this study. It contains the following 9 metrics at the county levels in the United States with dailyincluding average person-miles traveled on all traffic modes, average number of trips taken per person, percentage of residents staying at home (i.e., no trips more than one mile away from home), the percent of all trips taken that travel out of a county, number of work trips per person, percentage of workforce working from home, unemploy rate weekly and cumulative inflation rate and percentage of change in consumptionWe collected daily mobility metrics during January 1, 2020 and May, 24, 2020 from the platform.

### 2.3 Crash data

Our crash data occupancy were obtained from Finest Online Records Management System (FORMS), which is maintained by the New York State Department of Transportation (NYDOT) [14]. People invovled in the crashes is selected as dependent variable that means person (driver, occupant, pedestrian, bicyclist etc.) involved in a crash (see S1 Table). The crash report (MV104-AN) is required to be filled out for collisions where someone is injuryed or killed or where there is at least $1000 worth of damage [14].

### 2.4 Control policies implementation during pandemic from February 26-May 30, 2020

In this study, we collected the tpyes and timing of public policies issued by government using Google searches for new media coverage of local COVID-19 orders and proclamations followed by searching New York city government websites. Confirmed cumulative COVID-19 case count data were collected from USA Facts [15], which aggregates data on cases by date of the report from New York government.

Finally, the mobility, crash report, social and economic data for model development are presented in Table 1. Table 2 shows that Public policies ordering COVID-19 community mitigation interventions and dates of issuance in New York City, February 26-May 30, 2020. For all of the policies mentioned in Table 2, 1 represents the corresponding policy was implemented on a certain day, and 0 represents the policy was not implemented on that day.

**Table 1:** Variables considered for the model.

Variables	Mean	SD	Min	Max
**Dependent variable**				
Daily number of person invovled in a crash during Jan. 1^st^ -24^th^, 2020	43.89	25.54	11	103
**Exposure variables**				
Miles traveled/person	22.74	0	7.3	41.8
**Explanatory variables**				
***Mobility metrics***				
Average number of trips taken per person.	2.32	0.55	1.29	3.27
Percentage of residents staying at home (i.e., no trips more than one mile away from home)	52.86	39	36	75
The percent of all trips taken that travel out of a county.	14.44	7.76	6.5	22.30
Number of work trips per person (where a “work trip” is defined as going to or coming home from work)	0.43	0.15	0.23	0.76
***Social and economic factors***				
Percentage of workforce working from home.	18.63	12.34	4.2	40.2
Unemploy rate weekly.	11.42	9.83	3.7	29.8
Overall economic condition measured by cumulative inflation rate since COVID-19 outbreak.	0.53	0.41	-0.21	1
% change in consumption from the pre-pandemic baseline based on observed changes in trips to various types of consumption sites as a proxy.	-17.38	18.78	-54.1	15.9

**Table 2:** Public policies ordering COVID-19 community mitigation interventions and dates of issuance in New York City, February 26-May 30, 2020.

State (Main city)	Policy Variable (Symbol)	Social distancing (Start time)
New York City	P1	State declaration of emergency. (March 7)
	P2	Limits on mass gatherings. (March 12)
	P3	Stay at home order. (March 20)
	P4	Ban on all nonessential gathering. (March 20)

## 3. Methods

We modeled the number of people involved in crashes consistent with previous studies [16–33]. The Negative Binomial modeling technique was used to model the frequency of person invovled in crashes by specifying:

$$ln\lambda_{i}=\beta x_{i}+\varepsilon$$ (1)

Where *λ*_*i*_ is the expected mean number of person (driver, occupant, pedestrian, bicyclist) invovled in crashes on the ith day of the New York City; β is the vector representing parameters to be estimated; x_i_ is the vector representing the explanatory variables on the ith day including daily mobility metrics, policy implementation, social and economic variables; ε is the error term, where exp(ε) has a gamma distribution with mean 1 and variance *α*^2^.

The resulting probability distribution is as follows:

$$Prob(n_{i}|\varepsilon )=\frac{\exp\left[-\lambda_{i}\exp\left(\varepsilon \right)\right]\lambda_{i}^{n_{i}}}{n_{i}!}$$ (2)

Where, n_i_ is the number of person (driver, occupant, pedestrian, bicyclist) ivolved in crashes in New York City over a time period i.e., Jan. 1^st^, to May 24^th^, 2020. Integrating ε out of this expression produces the unconditional distribution of n_i_. The formulation of this distribution is:

$$Prob(n_{i})=\frac{\Gamma (\theta +n_{i})}{\left(\Gamma (\theta )n_{i}!\right)}\mu_{i}^{\theta }{\left(1-\mu_{i}\right)}^{n_{i}}$$ (3)

Where $\mu_{i}=\frac{\theta }{\theta +\lambda_{i}}$ and $\theta =\frac{1}{\alpha }$.

The Negative Binomial model can be estimated by standard maximum likelihood methods [23]. The corresponding likelihood function is:

$$L(\lambda_{i})=\prod_{i=1}^{N}\frac{\Gamma (\theta +n_{i})}{\left(\Gamma (\theta )n_{i}!\right)}\mu_{i}^{\theta }{\left(1-\mu_{i}\right)}^{n_{i}}$$ (4)

Where N is the total number days of COVID-19 pandemic period for our data set. This function is maximized to obtain coefficient estimates for *β* and *α*.

## 4. Results analysis

The Negative Binomial results for the number of person (driver, occupant, pedestrian, bicyclist) involved crashes are presented in Table 3. This table shows that all the variables have the expected sign (with a positive sign indicating an increase in the crashes frequency and a negative sign indicating a decrease).

**Table 3:** Negative binomial model of person involved in crash during COVID-19 pandemic.

	Coefficient	Std. Error	P-Value
Log (Miles traveled/person)	0.50	0.13	<0.01
Policy 3	-0.47	0.16	<0.01
Log (Number of work trips per person)	0.41	0.11	<0.01
Log (Unemployment rate weekly)	-0.30	0.11	<0.01
Log (Overall economic condition measured by cumulative inflation rate since COVID-19 outbreak.)	-0.44	0.18	0.02
Intercept	3.79	0.49	<0.01

As Table 3 shows, five variables had a significant association with the number of people involved in crashes: average person-miles traveled on all modes, work trip percentage, unemployment rate, inflation rate and stay at home order. The first is the exposure variables i.e., the log of the average person-miles traveled on all modes (car, train, bus, plane, bike, walk, etc.). An increase in person-miles traveled on all traffic modes has a positive impact on the number of people involved in crashes, with coefficients estimated at 0.5. The positive relationship between person-miles traveled and the number of people involved in crashes found in our study is very likely attributable to one reason. As walking trip and motor vehicle miles travelled decrease, the absolute number of people involved in crashes also decreases.

The percentage of work trip is significant and positive. Crashes increase with the increase of the work trip in New York City during COVID-19 outbreak. On the contrary, with respect to the variables related to social and economic characteristics, the log of unemployment rate and inflation rate have negative effects on the number of people involved crash, with coefficients estimated at -0.3 and -0.44, respectively.

For the four control measures implemented in New York City during COVID-19 pandemic, we take these four control policies as explanatory factors to evaluate the number of people involved in crashes. Different level of control policies during COVID-19 outbreak are closely associated with safety awareness, driving and travel behavior, and thus may have an indirect influence on the frequency of crashes. Comparing to the other three control policies including emergence declare, limits on mass gatherings, and ban on all nonessential gathering, the negative relationship between stay at home policy implemented in New York City since March 20, 2020 and the number of people involved crashes is found in our study.

**Fig 2:**
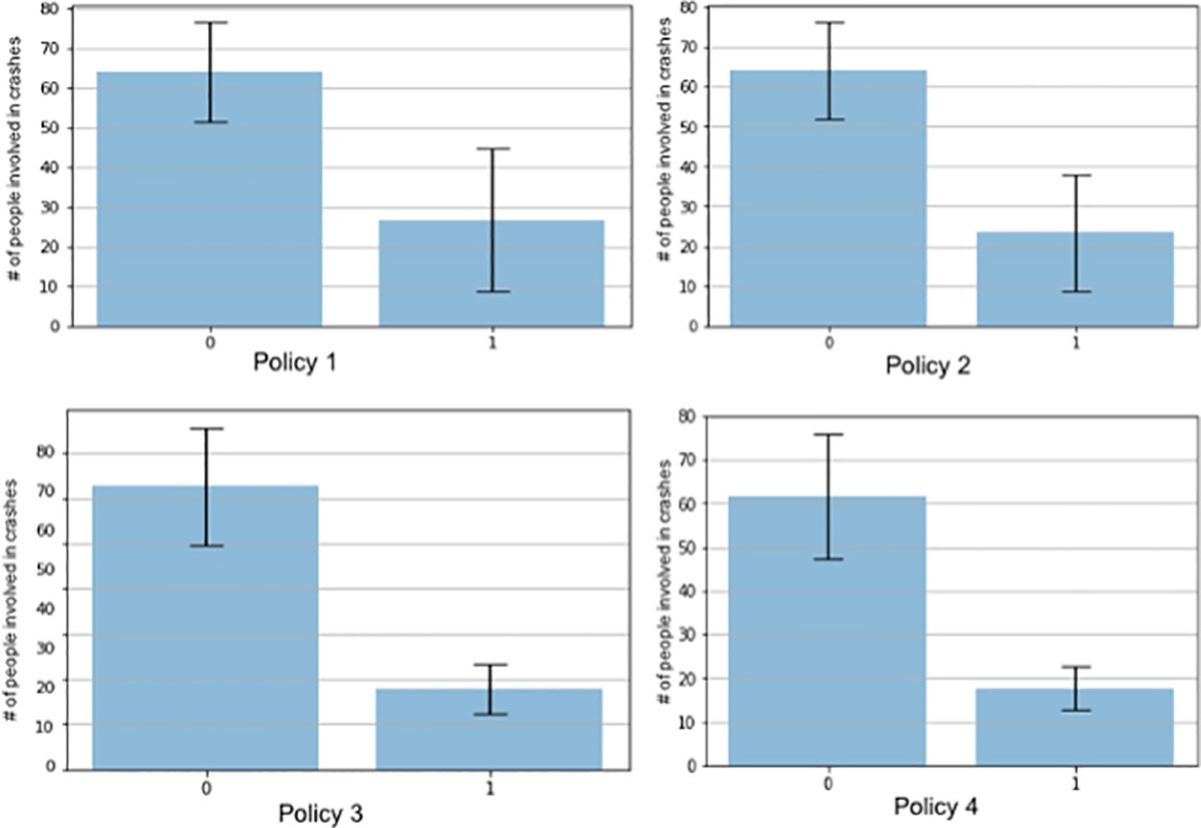
T-test results for four different control policies implemented in New York City during COVID-19 pandemic.

Finally, t-test is used to determine whether there is a significant different impact on traffic safety during COVID-19 between these four control measures. The results represent the impact of different control policies on the number of people involved in crashes. As Fig 2. Shows, all the policies have significant impact on the number of people involved in crashes (p-value<0.01). Meanwhile, stay at home and ban on all nonessential gathering have stronger influence on person involved in crashes. One of the possible reasons is these two control police further reduce the number of trips, which impact on the number of people involved in crashes.

## 5. Conclusion

This paper investigates the effect of human mobility and control policies including emergency declarations, bans on gatherings of certain sizes, school closures, restrictions on businesses, and stay-at-home or shelter-in-place of residence orders on the number of persons involved in crashes in New York City during COVID-19 pandemic. New York City was chosen as the site for our case study, since the city suffered from the first wave of COVID-19 spread during March and April and has a high volume of traffic. Meanwhile, a significant amount of data could be generated from the mobile device to collect residents’ trip information. These factors combined to outline a vivid picture of how this pandemic has impacted person involved in crashes.

Based on a comprehensive dataset of people involved in crashes aggregated and mobile device data in New York City between January 1, 2020 and May 24, 2020, we capture the impact of COVID-19 on traffic safety are in our study. According to our results, five variables were ultimately found to have a significant association with the number of people involved in crashes average person-miles traveled on all modes, work trip percentage, unemployment rate, inflation rate and stay at home order. The log of unemployment rate has negative effects on the number of people involved crash in our study. With more people stay at home reducing in the use of motor vehicles during the COVID-19 pandemic, the number of people involved in crashes decreased. It implied that some countermeasures might include restricting the use of motor vehicles and promotion of a shift from motor vehicles to walking for walkable-distance trip.

As of August 14, all of New York’s 10 regions are in the fourth and theoretically final phase of a four-phase reopening process following the statewide coronavirus lockdown; New York City was the last the enter Phase Four on July 20, while every other region had been in Phase Four for weeks, many new challenges faced by transportation systems remain. As the world continues to adjust to the new reality of COVID-19, there is a clear behavioral change including shared use modes to driving, walking, and biking, some employment industries taking more car trips to work etc. In future work, we can predict the effects of human mobility pattern under COVID-19 reopening scenarios on crash occurrence. At the same time, we will also develop models for crash spatial and temporal analysis using more complete methods.

## Supporting information

S1 Table The information is including the motor vehicle collisions person table contains details for people involved in the crash and the COVID-19 impact analysis data currently contains the following 9 metrics at the county levels in the United States with daily updates COVID-19 impact analysis data currently contains the following 9 metrics at the county levels in the United States with daily updates the COVID-19 impact analysis data currently contains the following 9 metrics at the county levels in the United States with daily updates. (DOCX)
