# Supplementary material for: The effect of human mobility and control measures on traffic safety during COVID-19 pandemic
Source: PLoS One. 2021 Mar 8;16(3):e0243263. doi: 10.1371/journal.pone.0243263 (PMC7939376; doi:10.1371/journal.pone.0243263)
Supplement: S1 Table — (DOCX) [file pone.0243263.s001.docx]

**S1 Table.** The information is including the Motor Vehicle Collisions person table contains details for people involved in the crash and the COVID-19 impact analysis data currently contains the following 9 metrics at the county levels in the United States with daily updates COVID-19 impact analysis data currently contains the following 9 metrics at the county levels in the United States with daily updates the COVID-19 impact analysis data currently contains the following 9 metrics at the county levels in the United States with daily updates.

|  | CRASH_DATE | frequency | Miles/person | Work trips/person | Unemployment rate rank | % change in consumption rank |
| --- | --- | --- | --- | --- | --- | --- |
| 1 | 1/1/2020 | 52 | 36.3 | 0.3 | 77 | 48 |
| 2 | 1/2/2020 | 51 | 31.3 | 0.56 | 77 | 30 |
| 3 | 1/3/2020 | 56 | 32.6 | 0.55 | 77 | 70 |
| 4 | 1/4/2020 | 43 | 34 | 0.36 | 77 | 47 |
| 5 | 1/5/2020 | 35 | 34.4 | 0.34 | 77 | 48 |
| 6 | 1/6/2020 | 55 | 30.1 | 0.61 | 77 | 82 |
| 7 | 1/7/2020 | 57 | 25.8 | 0.62 | 77 | 65 |
| 8 | 1/8/2020 | 61 | 30.3 | 0.63 | 77 | 79 |
| 9 | 1/9/2020 | 72 | 26.5 | 0.63 | 77 | 81 |
| 10 | 1/10/2020 | 70 | 29.8 | 0.63 | 77 | 52 |
| 11 | 1/11/2020 | 62 | 30.3 | 0.39 | 77 | 8 |
| 12 | 1/12/2020 | 52 | 33.3 | 0.38 | 77 | 27 |
| 13 | 1/13/2020 | 63 | 29.2 | 0.57 | 77 | 57 |
| 14 | 1/14/2020 | 64 | 26.6 | 0.56 | 77 | 74 |
| 15 | 1/15/2020 | 67 | 27.2 | 0.58 | 77 | 74 |
| 16 | 1/16/2020 | 65 | 31.1 | 0.6 | 77 | 55 |
| 17 | 1/17/2020 | 89 | 39.3 | 0.57 | 77 | 37 |
| 18 | 1/18/2020 | 99 | 30 | 0.36 | 77 | 61 |
| 19 | 1/19/2020 | 57 | 39.4 | 0.35 | 77 | 16 |
| 20 | 1/20/2020 | 58 | 38.5 | 0.41 | 77 | 94 |
| 21 | 1/21/2020 | 78 | 30.8 | 0.6 | 77 | 72 |
| 22 | 1/22/2020 | 64 | 29.1 | 0.59 | 77 | 71 |
| 23 | 1/23/2020 | 63 | 35.8 | 0.63 | 77 | 37 |
| 24 | 1/24/2020 | 55 | 32.7 | 0.6 | 77 | 48 |
| 25 | 1/25/2020 | 58 | 28.4 | 0.35 | 77 | 81 |
| 26 | 1/26/2020 | 59 | 33.7 | 0.36 | 77 | 31 |
| 27 | 1/27/2020 | 66 | 28.8 | 0.61 | 77 | 61 |
| 28 | 1/28/2020 | 49 | 29.3 | 0.61 | 77 | 63 |
| 29 | 1/29/2020 | 57 | 26.9 | 0.64 | 77 | 56 |
| 30 | 1/30/2020 | 71 | 30 | 0.63 | 77 | 38 |
| 31 | 1/31/2020 | 62 | 37 | 0.59 | 77 | 50 |
| 32 | 2/1/2020 | 51 | 27.7 | 0.4 | 77 | 48 |
| 33 | 2/2/2020 | 59 | 32.7 | 0.36 | 77 | 44 |
| 34 | 2/3/2020 | 81 | 32.7 | 0.6 | 77 | 62 |
| 35 | 2/4/2020 | 67 | 25.3 | 0.62 | 77 | 61 |
| 36 | 2/5/2020 | 71 | 30 | 0.63 | 77 | 44 |
| 37 | 2/6/2020 | 68 | 27.8 | 0.62 | 77 | 36 |
| 38 | 2/7/2020 | 82 | 29.6 | 0.61 | 77 | 53 |
| 39 | 2/8/2020 | 50 | 30.4 | 0.39 | 77 | 17 |
| 40 | 2/9/2020 | 57 | 28.5 | 0.33 | 77 | 34 |
| 41 | 2/10/2020 | 69 | 29.3 | 0.6 | 77 | 84 |
| 42 | 2/11/2020 | 72 | 25.6 | 0.62 | 77 | 73 |
| 43 | 2/12/2020 | 55 | 29.1 | 0.63 | 77 | 66 |
| 44 | 2/13/2020 | 60 | 31.8 | 0.63 | 77 | 46 |
| 45 | 2/14/2020 | 77 | 41.8 | 0.62 | 77 | 22 |
| 46 | 2/15/2020 | 79 | 34.7 | 0.37 | 77 | 33 |
| 47 | 2/16/2020 | 63 | 37.1 | 0.36 | 61 | 12 |
| 48 | 2/17/2020 | 46 | 38.6 | 0.41 | 61 | 45 |
| 49 | 2/18/2020 | 44 | 31.7 | 0.63 | 61 | 76 |
| 50 | 2/19/2020 | 58 | 33.1 | 0.64 | 61 | 67 |
| 51 | 2/20/2020 | 76 | 33.3 | 0.64 | 61 | 51 |
| 52 | 2/21/2020 | 56 | 38.3 | 0.61 | 61 | 64 |
| 53 | 2/22/2020 | 49 | 34.4 | 0.4 | 61 | 25 |
| 54 | 2/23/2020 | 55 | 36.9 | 0.39 | 61 | 22 |
| 55 | 2/24/2020 | 62 | 35.2 | 0.68 | 61 | 62 |
| 56 | 2/25/2020 | 66 | 29 | 0.68 | 61 | 82 |
| 57 | 2/26/2020 | 63 | 31.4 | 0.7 | 61 | 84 |
| 58 | 2/27/2020 | 81 | 31.9 | 0.73 | 61 | 81 |
| 59 | 2/28/2020 | 69 | 40.1 | 0.69 | 61 | 73 |
| 60 | 2/29/2020 | 57 | 40.4 | 0.42 | 61 | 63 |
| 61 | 3/1/2020 | 70 | 35.5 | 0.4 | 61 | 66 |
| 62 | 3/2/2020 | 89 | 29.3 | 0.73 | 61 | 85 |
| 63 | 3/3/2020 | 78 | 27.5 | 0.74 | 61 | 91 |
| 64 | 3/4/2020 | 65 | 26.7 | 0.76 | 61 | 80 |
| 65 | 3/5/2020 | 61 | 30.4 | 0.76 | 61 | 79 |
| 66 | 3/6/2020 | 103 | 33.9 | 0.68 | 61 | 86 |
| 67 | 3/7/2020 | 66 | 32.5 | 0.4 | 61 | 55 |
| 68 | 3/8/2020 | 52 | 36.7 | 0.38 | 61 | 49 |
| 69 | 3/9/2020 | 73 | 30.4 | 0.7 | 61 | 78 |
| 70 | 3/10/2020 | 56 | 23.5 | 0.68 | 61 | 94 |
| 71 | 3/11/2020 | 70 | 28.4 | 0.67 | 61 | 95 |
| 72 | 3/12/2020 | 69 | 28.1 | 0.65 | 61 | 96 |
| 73 | 3/13/2020 | 61 | 27.8 | 0.61 | 61 | 94 |
| 74 | 3/14/2020 | 59 | 30.7 | 0.38 | 61 | 65 |
| 75 | 3/15/2020 | 48 | 26.3 | 0.35 | 38 | 81 |
| 76 | 3/16/2020 | 58 | 23.2 | 0.52 | 38 | 99 |
| 77 | 3/17/2020 | 59 | 25.3 | 0.49 | 38 | 99 |
| 78 | 3/18/2020 | 21 | 18.9 | 0.48 | 38 | 99 |
| 79 | 3/19/2020 | 44 | 19.6 | 0.45 | 38 | 99 |
| 80 | 3/20/2020 | 49 | 17.5 | 0.43 | 38 | 98 |
| 81 | 3/21/2020 | 33 | 17 | 0.3 | 38 | 95 |
| 82 | 3/22/2020 | 11 | 15.4 | 0.27 | 65 | 96 |
| 83 | 3/23/2020 | 22 | 13.2 | 0.34 | 65 | 100 |
| 84 | 3/24/2020 | 18 | 11.3 | 0.36 | 65 | 99 |
| 85 | 3/25/2020 | 18 | 11.6 | 0.35 | 65 | 100 |
| 86 | 3/26/2020 | 23 | 12.7 | 0.36 | 65 | 99 |
| 87 | 3/27/2020 | 25 | 11.7 | 0.34 | 65 | 99 |
| 88 | 3/28/2020 | 25 | 14.4 | 0.24 | 65 | 99 |
| 89 | 3/29/2020 | 18 | 12.9 | 0.24 | 63 | 99 |
| 90 | 3/30/2020 | 14 | 11.3 | 0.32 | 63 | 99 |
| 91 | 3/31/2020 | 25 | 9.3 | 0.34 | 63 | 100 |
| 92 | 4/1/2020 | 17 | 9.2 | 0.34 | 63 | 99 |
| 93 | 4/2/2020 | 21 | 9.2 | 0.33 | 63 | 99 |
| 94 | 4/3/2020 | 17 | 8.4 | 0.33 | 63 | 99 |
| 95 | 4/4/2020 | 13 | 11.3 | 0.27 | 63 | 97 |
| 96 | 4/5/2020 | 13 | 9.3 | 0.26 | 67 | 97 |
| 97 | 4/6/2020 | 19 | 9.9 | 0.32 | 67 | 100 |
| 98 | 4/7/2020 | 20 | 10.7 | 0.35 | 67 | 99 |
| 99 | 4/8/2020 | 14 | 10 | 0.34 | 67 | 99 |
| 100 | 4/9/2020 | 15 | 9.6 | 0.32 | 67 | 99 |
| 101 | 4/10/2020 | 14 | 8.7 | 0.3 | 67 | 100 |
| 102 | 4/11/2020 | 11 | 10.5 | 0.25 | 67 | 98 |
| 103 | 4/12/2020 | 11 | 8.7 | 0.23 | 79 | 88 |
| 104 | 4/13/2020 | 17 | 7.3 | 0.3 | 79 | 99 |
| 105 | 4/14/2020 | 13 | 8.8 | 0.35 | 79 | 99 |
| 106 | 4/15/2020 | 24 | 11 | 0.34 | 79 | 99 |
| 107 | 4/16/2020 | 19 | 10.2 | 0.32 | 79 | 99 |
| 108 | 4/17/2020 | 21 | 9.2 | 0.33 | 79 | 99 |
| 109 | 4/18/2020 | 17 | 9.8 | 0.24 | 79 | 99 |
| 110 | 4/19/2020 | 10 | 12.8 | 0.24 | 68 | 92 |
| 111 | 4/20/2020 | 20 | 8.5 | 0.32 | 68 | 100 |
| 112 | 4/21/2020 | 11 | 10.5 | 0.33 | 68 | 100 |
| 113 | 4/22/2020 | 18 | 10.8 | 0.32 | 68 | 100 |
| 114 | 4/23/2020 | 13 | 10.4 | 0.33 | 68 | 100 |
| 115 | 4/24/2020 | 17 | 10.4 | 0.3 | 68 | 100 |
| 116 | 4/25/2020 | 14 | 13.3 | 0.27 | 68 | 95 |
| 117 | 4/26/2020 | 15 | 10.7 | 0.23 | 69 | 99 |
| 118 | 4/27/2020 | 15 | 9.4 | 0.31 | 69 | 100 |
| 119 | 4/28/2020 | 15 | 11.2 | 0.32 | 69 | 99 |
| 120 | 4/29/2020 | 8 | 11.5 | 0.33 | 69 | 100 |
| 121 | 4/30/2020 | 15 | 10.6 | 0.32 | 69 | 100 |
| 122 | 5/1/2020 | 29 | 14.5 | 0.32 | 69 | 100 |
| 123 | 5/2/2020 | 24 | 14.5 | 0.27 | 69 | 95 |
| 124 | 5/3/2020 | 16 | 13 | 0.26 | 72 | 94 |
| 125 | 5/4/2020 | 29 | 12.1 | 0.32 | 72 | 99 |
| 126 | 5/5/2020 | 22 | 13.7 | 0.33 | 72 | 99 |
| 127 | 5/7/2020 | 1 | 11.3 | 0.33 | 72 | 100 |
| 128 | 5/8/2020 | 2 | 12.8 | 0.35 | 72 | 99 |
| 129 | 5/9/2020 | 2 | 18.1 | 0.35 | 72 | 100 |
| 130 | 5/12/2020 | 1 | 16.2 | 0.26 | 72 | 99 |
| 131 | 5/13/2020 | 5 | 16.4 | 0.26 | 76 | 93 |
| 132 | 5/14/2020 | 2 | 14.7 | 0.33 | 76 | 99 |
| 133 | 5/15/2020 | 2 | 13 | 0.33 | 76 | 99 |
